# Detonation Velocity Measurement with Chirped Fiber Bragg Grating

**DOI:** 10.3390/s17112552

**Published:** 2017-11-06

**Authors:** Peng Wei, Hao Lang, Taolin Liu, Dong Xia

**Affiliations:** School of Instrument Science and Opto-Electronics Technology, Beihang University, Beijing 100191, China; weipeng@buaa.edu.cn (P.W.); liutl1992@163.com (T.L.); summereast@buaa.edu.cn (D.X.)

**Keywords:** chirped fiber Bragg grating, detonation velocity, relative standard uncertainty

## Abstract

Detonation velocity is an important parameter for explosive, and it is crucial for many fields such as dynamic chemistry burn models, detonation propagation prediction, explosive performance estimation, and so on. Dual-channel detonation velocity measurement method and system are described. The CFBG sensors are pasted both on the surface and in the center of the explosive cylinder. The length of CFBG sensors is measured via the hot-tip probe method. The light intensity reflected from the CFBG sensors attached to the explosive is transformed to voltage, and the voltage–time is then measured with the oscilloscope. According to the five experiments results, the relative standard uncertainty of detonation velocity is below 1%.

## 1. Introduction

A detonation wave is a shock wave with an intense chemical reaction and propagates in explosives. Detonation velocity can reach several kilometers per second. It is hardly affected by the outside environment and is a critical parameter for estimating explosive performance. It is significant for explosive proportion studies, explosion control, explosive equipment improvements, hydrocode calibration, dynamic chemistry burn models, and initiation and detonation processes [1].

There are some conventional ways of measuring detonation velocity, such as electrical shorting pins, microwave interferometry, high-speed photography, and PDV (photonic Doppler velocimetry) [2]. However, these approaches retain some drawbacks. Microwave interferometry [3] is a method that can get the continuous velocity by analyzing the beat signal, which is a superposition of the return signal from the detonation wavefront and the reference signal. However, it can be only used to measure the detonation velocity in linear waveguide materials, rather than metal. Electrical pins have been used for many years because of its ease of use and high accuracy. However, they can only be used to measure the average velocity between pins and obtain discreet datasets. This method has no ability in anti-electromagnetic interference. Owing to its high expense and complex operation, high-speed photography is also unsatisfied. The latest velocimetry [4,5] approach, i.e., PDV, is hard to measure at ultrafast speeds and is prohibitively expensive. Although it can obtain a continuous velocity, it requires great care to make sure that the embedded PDV probe is parallel to the detonation wave propagating direction. Additionally, the fiber probe needs to be assembled in a 1.6 mm diameter Teflon tube, which will have an influence on detonation wave propagation due to its large size [6].

The chirped fiber Bragg grating (CFBG) approach was developed by Eric Udd at McDonnell Douglas and Blue Road Research in 2004 and was used to measure the shock velocity in water [7]. Then, Jerry Benterou at Columbia Gorge Research and Eric Udd made great improvements to this method [8]. G. Rodriguez at the Los Alamos National Laboratory has conducted a series of studies to measure the detonation velocity of different materials including PBX 9501, PBX 9502, Comp B, TNT, PBX 9407, PBX 9520, PMMA(polymethyl methacrylate), and so on [9,10].

CFBGs are fiber sensors. CFBGs offer an attractive alternative means for measuring detonation velocity. They can track velocity changes across interfaces of different explosive compositions and can be used in liquid explosives or metals. Their small diameters (125 um) enable in situ detonation velocity measurement by embedding the CFBG directly inside an explosive [11].

In this paper, a detonation velocity method using CFBGs was described. We set up a dual-channel CFBG velocimetry and measured the detonation velocity inside a certain explosive cylinder and along its length on the surface at the same time. We performed five experiments. By analyzing the experimental data, we obtained the average velocity and relative standard uncertainty. The relative uncertainty of velocity is below 1%.

## 2. Methods

### 2.1. CFBG Sensor Selection

The index of refraction in the core of a CFBG sensor varies periodically axially along the fiber grating. Therefore, there is a relationship between wavelength of light reflected from the CFBG and the grating position [12].

The Bragg wavelength equation is

(1) $$\lambda_{B}(z)=2n_{eff}\Lambda (z)$$

where $n_{eff}$ is the effective refractive index of the CFBG, Λ(z) is the period of the grating, and z is the position along the grating.

**Figure 1:**
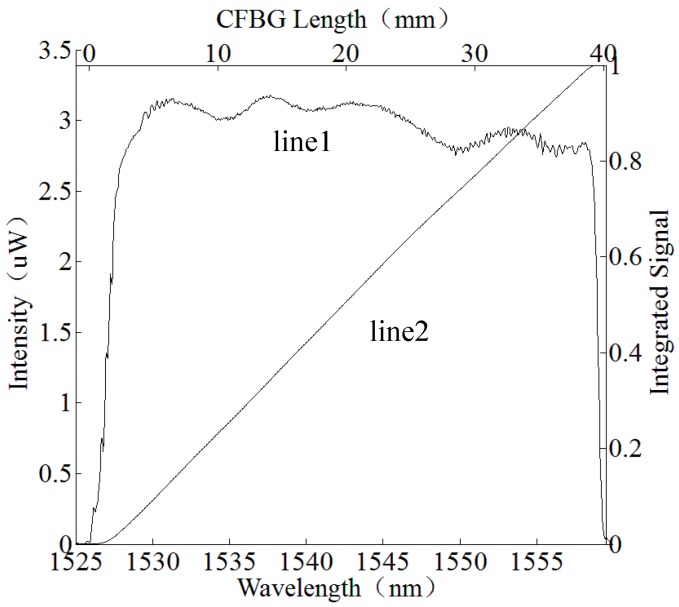
Line 1 is the spectrum of a chirped fiber Bragg grating (CFBG) reflected light. Line 2 is the numerical integration of the spectrum.

A linear CFBG is one kind of CFBG and its period changes linearly along the grating, shown as

(2) $$\Lambda (z)=\Lambda_{0}+Cz$$

where $\Lambda_{0}$ is the initial period on the beginning of the CFBG, and C is the chirp rate of the grating. One of the linear CFBGs is shown in Figure 1: the starting wavelength is 1525 nm, the ending wavelength is 1560 nm, and between them the wavelength is linearly increasing. In this paper, we use a linear CFBG.

Thus, a CFBG reflects a wide band of optical spectrum and the bandwidth of the reflected spectrum is

(3) $$\Delta \lambda =2n_{eff}[\Lambda (z_{end})-\Lambda (z_{0})]=2n_{eff}CL$$

where $z_{0}$ and $z_{end}$ represent the beginning and end positions of the CFBG, respectively, and L is the length of the CFBG.

In Figure 1, the reflection bandwidth of a CFBG is plotted as Line 1, Line 2 is a numerical integration of the reflection bandwidth. Since the wavelength of the CFBG reflection spectrum is linear in length (linear chirp), the CFBG has a nearly linear relationship between the length of the CFBG and the numerical integration of the reflection bandwidth.

**Figure 2:**
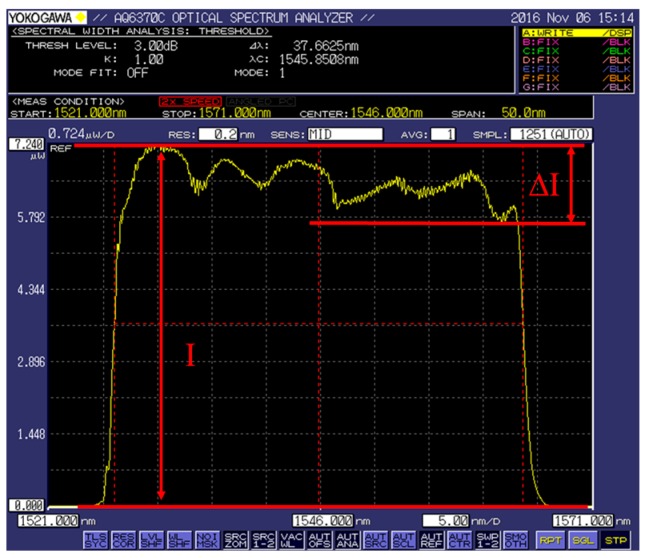
The spectrum of a CFBG reflected light. I is the maximum light intensity, and ΔI is the change range of light intensity on the top spectrum.

Figure 2 is the CFBG reflected spectrum. The maximum light intensity is I, and the change of light intensity on the top spectrum is ΔI. According to the theory of CFBGs and experimental experience, only those CFBG sensors whose ΔI/I values are below 20% meet the requirements and have good experiments results.

The bandwidth of the amplified spontaneous emission (ASE) source is usually between 1525 and 1565 nm. To simplify the experiment analysis, the reflection bandwidth of CFBG sensors should be in the ASE source spectral range so that only the flattest part of the spectrum is used.

According to experimental experience, the CFBG sensor with a higher chirped rate is more accurate in measuring the detonation velocity and mapping the changes across different explosive interfaces [13].

### 2.2. CFBG Length

**Figure 3:**
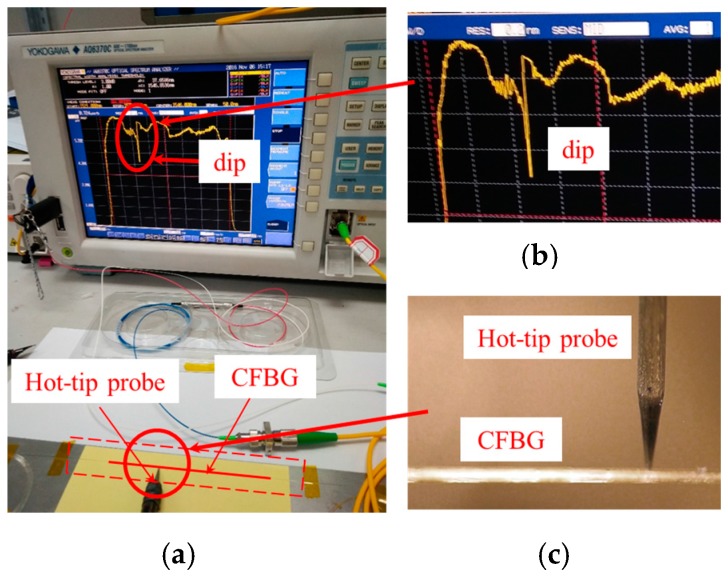
The calibration of CFBG length using the hot-tip method. (**a**) Setup. (**b**) The spectral dip when the hot-tip probe touches the grating. (**c**) Hot-tip probe and CFBG.

To calculate the detonation velocity, the CFBG length must be measured firstly and it has a vital influence on the velocity accuracy. The CFBG can be calibrated using a hot-tip micro-probe, which will not damage the CFBG. As Figure 3 shows, when the hot probe touches a certain position on the CFBG sensor, CFBG produces a temporary dip in the return spectrum. The spectrum returns to normal after removing the hot probe [7].

The position l that is touched by the hot probe and the corresponding wavelength λ of reflected spectrum is measured, and the relationship between position and the wavelength via linear fitting is obtained with the following:

(4) l=kλ+b

where k and b are slope and intercept parameters of the line.

Thus, the chirp rate C is 1/k.

**Figure 4:**
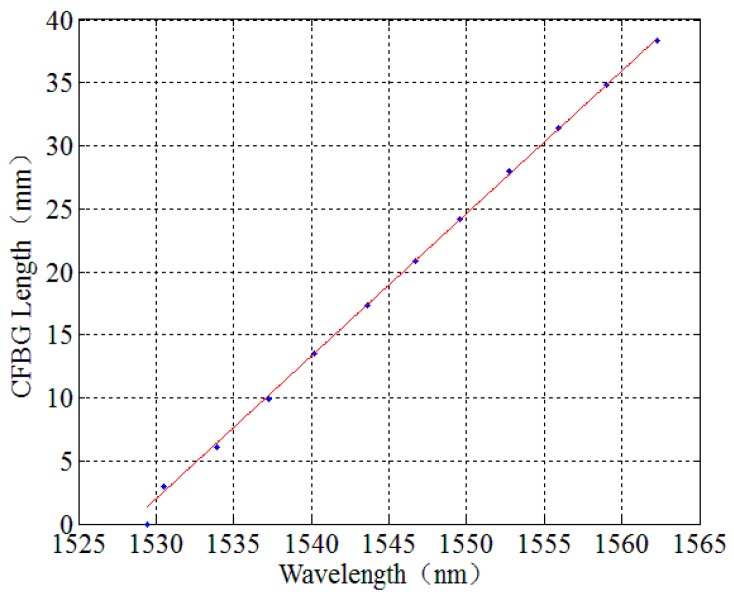
The relationship between CFBG wavelength and physical length. These data points were obtained with a hot-tip probe calibration method. The line was obtained by linear fitting using these data points.

The relationship about the wavelength and the length of a CFBG is plotted in Figure 4. The CFBG’s physical length is then calculated using the CFBG spectrum bandwidth Δλ, and it is shown below.

(5) ΔL=k⋅Δλ.

This non-destructive calibration is a simple-operation, low-cost, and high-accuracy approach [12].

### 2.3. CFBG Detonation Velocity Measurment

**Figure 5:**
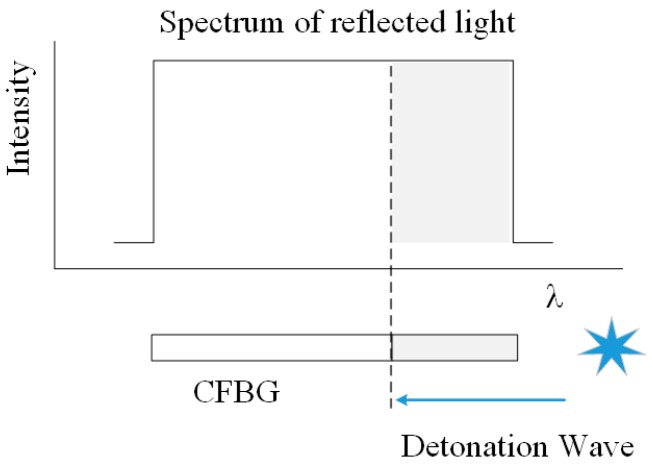
Scheme of the CFBG detonation velocity measurement principle.

The principle of CFBG detonation velocity measurement is illustrated in Figure 5. Light from the ASE source enters into the CFBG sensor through a 3-port power circulator [14]. The CFBG sensor structure will be damaged by the detonation wave when it propagates in the explosive and the CFBG has been damaged before the arrival of high temperature and pressure. Therefore, there is little influence of temperature and pressure on detonation velocity. As a consequence, the length of the CFBG will shorten, and some parts of the spectrum disappear. The reflection bandwidth of the grating becomes narrow, so the intensity of the reflected light decreases [15]. In Figure 5, the dashed area of the spectrum indicates the lost part, and the dashed part of the CFBG indicates the damaged area. The reflected light is directed into a fast InGasAs photodetector, and the light intensity is converted to a voltage recording by a fast digitizing oscilloscope. Owing to the linear relationship between the CFBG length and the numerical integration of the reflection bandwidth, the voltage has a linear relationship with the CFBG length. Therefore, the recorded trace of voltage versus time can be transformed to length versus time. Detonation velocity can be extracted by the length–time relationship [16].

Before the CFBG is destroyed by the detonation wave, the reflected spectrum is full. Assuming the response parameter of the detector is a constant, the voltage on the detector is proportional to the light intensity [17,18]. Therefore, the initial voltage before detonation ($Y_{max}$) is maximum. $Y_{norm}$ is the normalization of the maximum voltage shown as below

(6) $$Y_{norm}=1=K\int_{-\infty }^{+\infty }ASE(\lambda )R\left(\lambda \right)d\lambda$$

where ASE(λ) is the intensity of the ASE source, R(λ) is the reflectivity of the CFBG, and K is the normalized coefficient.

The length decreases and a fraction of the reflected light is lost as the gratin is consumed by the detonation wave. Therefore, the measured voltage is

(7) $$Y_{measured}=Y_{max}-K\int_{-\infty }^{\lambda *}ASE(\lambda )R\left(\lambda \right)d\lambda$$

where $\lambda^{*}$ is the wavelength position where the detonation wave is located.

Since the length is related to the voltage linearly, the CFBG length with time can be obtained as below:

(8) $$\;L(t)\;=\;\Delta L\cdot Y_{measured}$$

where ΔL is the length of the CFBG.

Therefore, the velocity is

(9) v(t) = dL(t)/dt.

## 3. Experiments and Results

### 3.1. Experiments

#### 3.1.1. Experiment Setup

**Figure 6:**
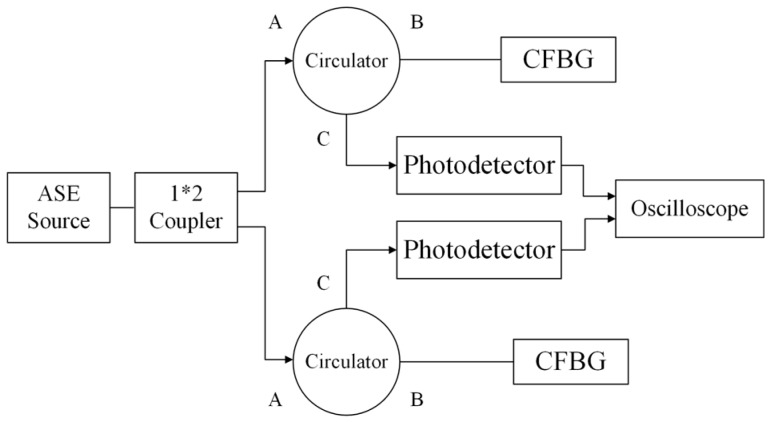
CFBG block diagram of dual-channel detonation velocity measurement system.

We set up a dual-channel CFBG detonation velocity measurement system. The system block diagram is shown in Figure 6. Using a 50:50 fiber coupler, the light is divided into two beams, and both of them then enter into two power circulators, respectively. Both of the photodetectors connect to the same oscilloscope.

The incoherent broad band ASE source centers around the C-band (1525–1565 nm). Its output power can be adjusted from 0 to 20 dBm, and the power stability is 0.02 dBm in 8 h. To simplify the data analysis procedure, the reflection bandwidth of the CFBG is shorter than the bandwidth of the light source.

The bandwidth of the InGasAs photo-detector is 10 MHz, the rise time is 80 ns, and the noise equivalent power is 0.8 $\mathrm{pW}/\sqrt{\mathrm{Hz}}$. The sampling rate of the oscilloscope is 5 Gs/s with an 8-bit sampling precision.

#### 3.1.2. CFBG Assembly

**Figure 7:**
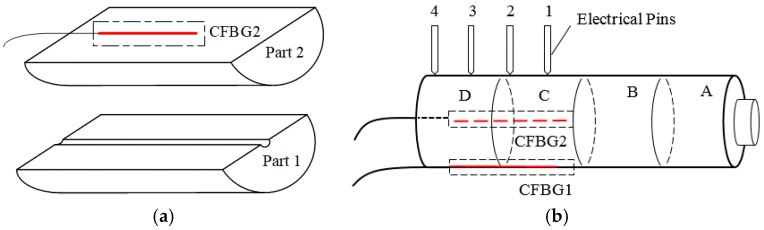
CFBG assembly diagram. (**a**) CBFG2 assembly process. (**b**) The whole structure diagram.

In the beginning, the explosive cylinder is composed of two parts. As Figure 7a shows, there is a small groove on Part 1 and the surface of Part 2 is plain. The CFBG2 was stuck on the flat surface of Part 2. Then, Part 1 and Part 2 was stuck together as shown in Figure 7b. The groove was filled by clay and CFBG2.

As illustrated by Figure 7b, the 43.05-mm-long CFBG1 (solid line) was glued on the explosive surface, and the 40.14-mm-long CFBG2 (dashed line) was glued in the coaxial center of the explosive cylinder. They were mounted parallel to the axial direction of the explosive cylinders. B, C and D are the same size (50 mm in diameter and 30 mm in length). Numbers 1–4 indicate electrical pins, and the distance between them is 15 mm.

### 3.2. Results

#### 3.2.1. Average Velocity

**Figure 8:**
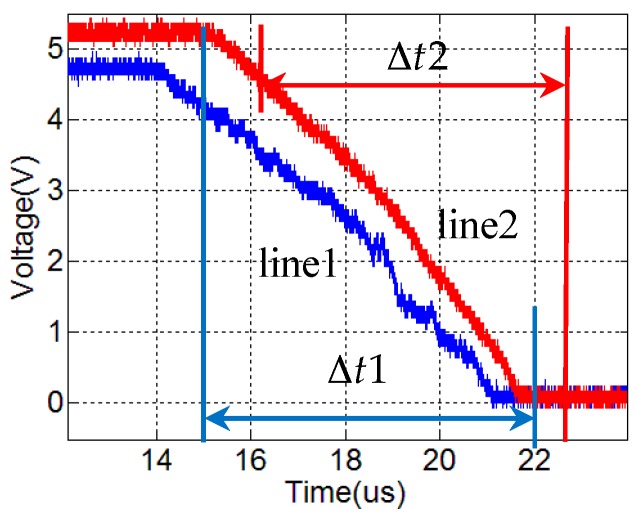
Raw data voltage versus time traces. Line 1 is CFBG1 data. Line 2 is CFBG2 data.

The raw voltage–time data is plotted in Figure 8. Line 1 is the data of CFBG1, while Line 2 is the data of CFBG2. The voltage of CFBG1 before detonation is 4.96 V, and the voltage of CFBG2 is 5.28 V. Eventually, the voltage decreased to 0 V when the explosion ends.

The destroying time of CFBG1 is 7.16 us, so the average velocity is

(10) $$v1=\frac{\Delta L1}{\Delta t1}=\frac{43.04\times 10^{-3}}{7.16\times 10^{-6}}=6011\;m/s.$$

The destroying time of CFBG2 is 6.53 us, so the average velocity is

(11) $$v2=\frac{\Delta L2}{\Delta t2}=\frac{40.14\times 10^{-3}}{6.53\times 10^{-6}}=6147\;m/s.$$

A two-point measurement was made to compare with the results of the electrical pins method.

#### 3.2.2. Uncertainty of Velocity

Taking CFBG2 as an example, a velocity uncertainty calculating method is illustrated below.

##### The Uncertainty of Length

The uncertainty of the CFBG length is mainly based on the calibration.

In Table 1, 10 points are used to calibrate the length of the CFBG. Ten points is not the only choice for calibration. However, in our experiment, 10 points was sufficient for CFBG length accuracy.

Using the data from Table 1, we can obtain a function of CFBG length and wavelength via linear fitting based on Equation (4):

l=1.139λ−1741.

$u_{c}(l)$ is the uncertainty of l, including Type A and Type B standard uncertainty. n is the point number of linear fitting, and $W_{i}$ is the residual.

$$\begin{array}{l} W_{i}=l_{i}-(k\lambda_{i}+b)=0.2248\\ n=10. \end{array}$$

Thus, the type A standard uncertainty [19,20,21] of l is

$$u_{A}(l)=\sqrt{\frac{\sum W_{i}{}^{2}}{n-2}}=0.1676\;mm.$$

The type B standard uncertainty of l stems from the error of indication of the vernier caliper. The minimum scale of the vernier caliper is 0.02 mm, and is regarded as a uniform distribution. Thus, the type B standard uncertainty of l is

$$u_{B}(l)=\frac{0.02}{\sqrt{3}}=0.0115\;mm.$$

Thus, the combining uncertainty of l is

$$u_{c}(l)=\sqrt{u_{A}^{2}(l)+u_{B}^{2}(l)}=0.168\;mm.$$

The uncertainty of k is

$$u_{k}=u_{c}(l)\sqrt{1/\sum_{1}^{n}{\left(\lambda_{i}-\bar{\lambda }\right)}^{2}}=0.0059.$$

Because of $\Delta \lambda =\lambda_{2}-\lambda_{1}$,

$$u_{\Delta \lambda }=\sqrt{2}u_{\lambda_{2}}=\sqrt{2}u_{\lambda 1}=\sqrt{2}u_{\lambda }.$$

The uncertainty of λ consists of the type B standard uncertainty $u_{B1}(\lambda )$ and $u_{B2}(\lambda )$, which stem from the stability of indication and error of indication of optic spectrum analyzer, respectively. The wavelength stability of the spectrometer in 1 min is 0.005 nm and the wavelength precision is 0.02 nm, so

$$\begin{array}{l} u_{B1}(\lambda )=0.005/\sqrt{3}=0.0028\;\mathrm{nm}\\ u_{B2}(\lambda )=0.02/\sqrt{3}=0.0115\;\mathrm{nm}. \end{array}$$

The uncertainty of λ and Δλ is shown below:

$$u_{\lambda }=\sqrt{u_{B1}^{2}(\lambda )+u_{B2}^{2}(\lambda )}=0.012\;\mathrm{nm}$$

$$u_{\Delta \lambda }=0.0168\;\mathrm{nm}.$$

Since ΔL=k⋅Δλ, the uncertainty of ΔL is

$$u_{\Delta L}=\sqrt{{\left(\frac{\partial \Delta L}{\partial \lambda }u_{\lambda }\right)}^{2}+{\left(\frac{\partial \Delta L}{\partial k}u_{k}\right)}^{2}}=\sqrt{{\left(ku_{\Delta \lambda }\right)}^{2}+{\left(\Delta \lambda u_{k}\right)}^{2}}=0.21\;\mathrm{mm}.$$

##### Time Uncertainty

The uncertainty of time consists in the type B standard uncertainty $u_{B1}(t)$ and $u_{B2}(t)$, which come from the error of indication of the digital oscilloscope and reading error, respectively. Since the time resolution of oscilloscope is 0.2 ns and reading error is 15 ns,

$$\begin{array}{l} u_{B1}(t)=0.2/\sqrt{3}=0.115\;\mathrm{ns}\\ u_{B2}(t)=15/\sqrt{3}=8.66\;\mathrm{ns}. \end{array}$$

Therefore, the combining uncertainty of time is

$$u_{\Delta t}=\sqrt{u^{2}{}_{B1}(t)+u^{2}{}_{B2}(t)}=8.66\;\mathrm{ns}.$$

##### Combining Uncertainty of Velocity

According to the function v=ΔL/Δt, ΔL and Δt are independent, so the standard uncertainty of velocity is

$$u_{v}=\sqrt{{\left(\frac{\partial v}{\partial \Delta L}u_{\Delta L}\right)}^{2}+{\left(\frac{\partial v}{\partial \Delta t}u_{\Delta t}\right)}^{2}}=\sqrt{{\left(\frac{1}{\Delta t}u_{\Delta L}\right)}^{2}+{\left(-\frac{\Delta L}{\Delta t^{2}}u_{\Delta t}\right)}^{2}}=42\;m/s.$$

At last, the velocity of detonation measured via CFBG2 is 6147 ± 42 m/s, and the relative standard uncertainty, which is the ratio of standard uncertainty to the measurements, is

42/6147≈0.683%.

In the same way, the velocity of detonation measured via CFBG1 is 6011 ± 48 m/s, and the relative standard uncertainty is 0.799%.

#### 3.2.3. Results of Five Experiments

In another four experiments, the CFBGs were mounted on the surface of the explosives.

The velocity results of all five experiments using the CFBG and electrical pins methods are shown in Table 2, as well as the results difference.

The relative standard uncertainty of all five experiments are shown in Table 3.

## 4. Discussion and Conclusions

From the results shown in the previous section, the relative standard uncertainty of detonation velocity using this CFBG method is below 1%, which means a good stability. Compared to the electrical results, the velocity measured via CFBGs shows little difference, which indicates high accuracy. Five different explosive samples were used in the experiments, and all of them led to good results, which indicates good reproducibility.

The velocity in the coaxial center of the explosive cylinder measured via CFBG2 is slightly higher than the CFBG1 result, while CFBG1 is on the surface of the explosive cylinder.

In this paper, a method of detonation velocity measurement using CFBG sensors is described. The velocity inside and alongside the explosive cylinder was obtained at the same time with the help of the CFBGs. Detonation velocity measurement using CFBG sensors is a novel and advantageous method. The characteristics of small size and flexibility of CFBGs enable the prospect of a quantitative in situ measurement because CFBGs can be assembled inside the explosive. Compared to the electrical pins method, this approach can be used in liquid explosives. This method has a unique potential to continuously track the velocity changes when the detonation travels through the interfaces between different explosive cylinders. In the future, the research should focus on differential and de-noising of the signal to obtain continuous velocity.

## Figures and Tables

**Table 1:** Wavelength and length data of the CFBG2.

Position	Wavelength/nm	Length/mm
1	1533.88	6.08
2	1537.24	9.90
3	1540.20	13.54
4	1543.60	17.32
5	1546.68	20.88
6	1549.60	24.16
7	1552.76	28.00
8	1555.92	31.44
9	1559.04	34.78
10	1562.28	38.32

**Table 2:** The velocity of the five experiments.

Experiment	CFBG Method (m/s)	Electrical Pins Method (m/s)	Difference ^1^
1	7072	6944	1.84%
2	6360	6288	1.15%
3	6180	6274	1.50%
4	6056	6162	1.72%
5(CFBG1)	6011	-	-
5(CFBG2)	6147	6226	1.27%

^1^ The difference of velocity measured via CFBG when compared to electrical pins results.

**Table 3:** The results of the experiments.

Experiment	Velocity (m/s)	Relative Standard Uncertainty
1	7072 ± 62	0.877%
2	6360 ± 56	0.881%
3	6180 ± 56	0.906%
4	6056 ± 52	0.859%
5(CFBG1)	6011 ± 48	0.799%
5(CFBG2)	6147 ± 42	0.683%

## Abbreviations

The following abbreviations are used in this manuscript.

CFBG	chirped fiber Bragg grating
PBX	plastic bonded explosive
ASE	amplified spontaneous emission
PDV	photonic Doppler velocimetry
